# Supplementary material for: New 5-Substituted SN38 Derivatives: A Stability Study and Interaction with Model Nicked DNA by NMR and Molecular Modeling Methods
Source: Int J Mol Sci. 2023 Dec 13;24(24):17445. doi: 10.3390/ijms242417445 (PMC10743537; doi:10.3390/ijms242417445)
Supplement: Supplementary file 1 [file ijms-24-17445-s001.zip › ijms-2711296-supplementary.pdf]

# Electronic Supplementary Materials

## New 5-substituted SN38 derivatives: A Stability Study and Interaction with Model nicked DNA by NMR and Molecular Modeling Methods

Elżbieta Bednarek [1,*], Wojciech Bocian [1], Jerzy Sitkowski [1], Magdalena Urbanowicz [1], Lech Kozerski [1]

[1] National Medicines Institute, Chełmska 30/34, 00-725 Warsaw, Poland

**Correspondence to Elżbieta Bednarek*

*National Medicines Institute 00-725 Warsaw, Chełmska 30/34*

*Phone +48 (22)8514372 ext 316 e-mail: e.bednarek@nil.gov.pl*

The structure of the compound 5(*R*)-(N-pyrrolidinyl)methyl-7-ethyl-10-hydroxycamptothecin (**1**) and its diastereomer 5(*S*) (**2**) obtained as formate salts was confirmed by NMR spectra analysis.

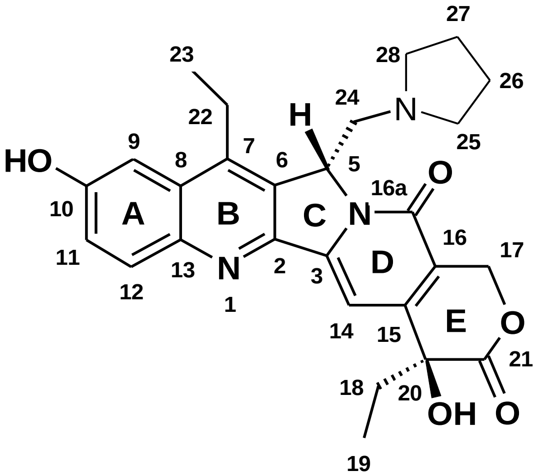

5(*R*)-(N-pyrrolidinyl)methyl-7-ethyl-10-hydroxycamptothecin x HCOOH (**1**)

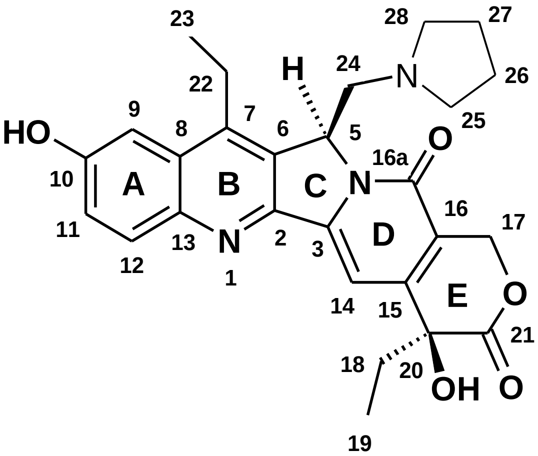

5(*S*)-(N-pyrrolidinyl)methyl-7-ethyl-10-hydroxycamptothecin x HCOOH (**2**)

The experimental $^{1}H$ and $^{13}C$ NMR chemical shifts for compounds **1** and **2** in $D_2O$/DMSO-*d6* solution, (90%/10%, pH 3) are presented in Table S1. Notably, the chemical shifts of proton signals for compounds **1** and **2** exhibited distinct differences, making it feasible to distinguish the configuration at C5 based on the $^{1}H$ NMR spectra. This distinction is particularly evident in the significant chemical shift changes observed for protons such as H12, H11, H14, and H5, with differences of 0.45, 0.17, 0.22, and 0.20 ppm, respectively. Furthermore, substantial differences in chemical shifts are also observed for proton signals from the (N-pyrrolidinyl)methyl substituent, including H24, H25, and H28, as well as for the H23 proton signal of the ethyl substituent at C7. Moreover, the magnetic nonequivalence of the protons in the C17-$H_2$ group is substantially greater for compound **2** (5*S*,20*S*) compared to **1** (5*R*,20*S*). Another distinguishing factor between diastereoisomers is the retention time, $R_f$, (see the HPLC chromatograms of compounds **1 (**$R_f$: 16.3 min) and **2 (**$R_f$: 17.6 min) in Figure S1)

**Table S1.** The experimental $^{1}$H and $^{13}$C NMR chemical shifts $\delta$ [ppm] for **1** (5*R*,20*S*) and **2** (5*S*,20*S*) in $D_2O$/DMSO-*d6*, 90%/10%, pH 3, temp. 25 °C (ref. to TSPA-$d_4$)

| **1** ( c= 1.39 mM) | | | **2** ( c= 1.67 mM) | | |
|---|---|---|---|---|---|
| Numbering of atom position | $\delta_{1H}$ * | $\delta_{13C}$ # | Numbering of atom position | $\delta_{1H}$ * | $\delta_{13C}$ # |
| **2** (C) | - | 149.32 (*H5, H14*) | **2** (C) | - | 150.40 (*H5, H14*) |
| **3** (C) | - | 148.04 (*H14, H17a*) | **3** (C) | - | 148.28 (*H5, H14, H17a*) |
| **5 (CH)** | 6.17 (*d*, 1H, *J* =7.9**)** | 63.16 (*H24a*) | **5 (CH)** | 6.372 (*d*, 1H, *J* =7.9) | 63.098 (*H24a*) |
| **6** (C) | - | 128.36 (*H5, H22, H24b*) | **6** (C) | - | 129.02 (*H5, H22, H24b*) |
| **7** (C) | - | 149.06 (*H9, H22, H23*) | **7** (C) | - | 149.28 (*H5, H9, H22, H23*) |
| **8** (C) | - | 131.75 (*H12, H22*) | **8** (C) | - | 131.77 (*H12, H22*) |
| **9 (CH)** | 7.478 (*d*, 1H, *J* =2.5) | 108.963 (*H11*) | **9 (CH)** | 7.511 (*d*, 1H, *J* =2.5) | 109.162 (*H11*) |
| **10** (C) | - | 158.895 (*H9, H12*) | **10** (C) | - | 158.803 (*H9, H12*) |
| **11**(CH) | 7.317 (*dd*, 1H, *J* =9.2, 2.5) | 126.23 (*H9*) | **11**(CH) | 7.4885 (*dd*, 1H, *J* = 9.1, 2.5) | 126.306 (*H9*) |
| **12** (CH) | 7.516 (*d*, 1H, *J* =9.2) | 133.25 | **12** (CH) | 7.968 (*d*, 1H, *J* = 9.1) | 133.445 |
| **13** (C) | - | 146.52 (*H9, H11*) | **13** (C) | - | 146.586 (*H9, H11*) |
| **14** (CH) | 7.329 (*s*, 1H) | 101.772 | **14** (CH) | 7.544 (*s*, 1H) | 102.356 (*H17a*) |
| **15** (C) | - | 154.44 (*H17a, H17b, H18*) | **15** (C) | - | 154.62 (*H17a, H17b, H18*) |
| **16** (C) | - | 122.0 (*H14, H17a, H17b*) | **16** (C) | - | 122.46 (*H14, H17a, H17b*) |
| **16a** (N-**C**=O) | - | 161.910 (*H17a, H17b*) | **16a** (N-**C**=O) | - | 162.44 (*H17a, H17b*) |
| **17a**<br>**17b** (CH$_2$) | 5.428 (*d*, 1H, *J* =16.5)<br>5.500 (*d*, 1H, *J* =16.5) | 68.508 | **17a**<br>**17b** (CH$_2$) | 5.425 (*d*, 1H, *J* = 16.2)<br>5.650 (*d*, 1H, *J* =16.2) | 68.617 |
| **18** (CH$_2$) | 1.989 (*m*, 2H) | 33.29 (*H19*) | **18** (CH$_2$) | 1.999 (*m*, 2H) | 33.412 (*H19*) |
| **19** (CH$_3$) | 0.955 (*t*, 3H, *J* =7.4) | 9.835 (*H18*) | **19** (CH$_3$) | 0.957 (*t*, 3H, *J* = 7.4) | 9.941 (*H18*) |
| **20** (C) | - | 76.26 (*H14, H18, H19*) | **20** (C) | - | 76.298 (*H14, H18, H19*) |
| **21** (O-**C**=O) | - | 177.42 (*H17b, H18*) | **21** (O-**C**=O ) | - | 177.352 (*H17b, H18*) |
| **22a**<br>**22b** (CH$_2$) | 3.182 (*m*, 2H)<br>3.261 (*m*, 2H) | 25.199 (*H23*) | **22** (CH$_2$) | 3.218 (*m*, 1H) | 25.181 (*H23*) |
| **23** (CH$_3$) | 1.524 (*t*, 3H, *J* =7.6) | 16.517 (*H22*) | **23** (CH$_3$) | 1.405 (*t*, 3H, *J* =7.6) | 16.440 (*H22*) |
| **24a**<br>**24b** (CH$_2$) | 3.698 (*dd*, 1H, *J* =14.8, 7.9)<br>3.920 (*d*, 1H, *J* =14.8) | 60.089 (*H5*) | **24a**<br>**24b** (CH$_2$) | 3.952 (*dd*, 1H, *J* =14.9, 7.9)<br>4.106 (*d*, 1H, *J* =14.9) | 60.049 (*H5*) |
| **25** (CH$_2$)$ | 3.013 (m, 1H);<br>3.520 (*brs*, 1H) | 58.642 (*H24a*) | **25** (CH$_2$)$ | 3.125 (m, 1H);<br>3.645 (*brs*, 1H) | 58.946 (*H24a*) |
| **26** (CH$_2$)& | 1.987 (*brs*, 1H);<br>2.140 (*brs*, 1H | 25.135 | **26** (CH$_2$)& | 2.028 (*brs*, 1H);<br>2.134 (*brs*, 1H | 25.237 |
| **27** (CH$_2$)& | 2.149 (*brs*, 1H);<br>2.268 (*brs*, 1H) | 25.744 | **27** (CH$_2$)& | 2.134 (*brs*, 1H);<br>2.254 (*brs*, 1H) | 25.768 |
| **28** (CH$_2$)$ | 3.341 (m, 1H);<br>4.274 (*brs*, 1H); | 57.45 (*H24a*) | **28** (CH$_2$)$ | 3.382 (m, 1H);<br>4.104 (*brs*, 1H); | 57.461 (*H24a*) |

* in brackets (multiplicity, number of protons, proton-proton coupling constants $J_{HH}$ [Hz]);

# in brackets the heteronuclear multiple bond diagnostic correlation between the given carbon atom and the showing proton(s) are presented;

$ the assignment in positions **25** and **28** can be interchanged; & the assignment in positions **26** and **27** can be interchanged;

*brs* broad singlet, *d* doublet, *dd* doublet of doublets, *m* multiplet, *s* singlet, *t* triplet, *q* quartet, *quint* quintet.

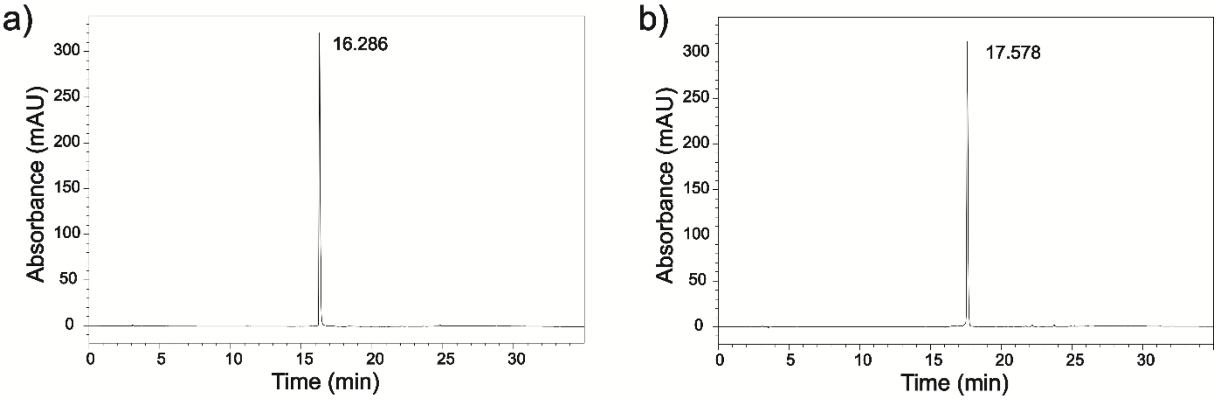


**Figure S1.** HPLC chromatograms of diastereomers: **1** (a) and **2** (b)

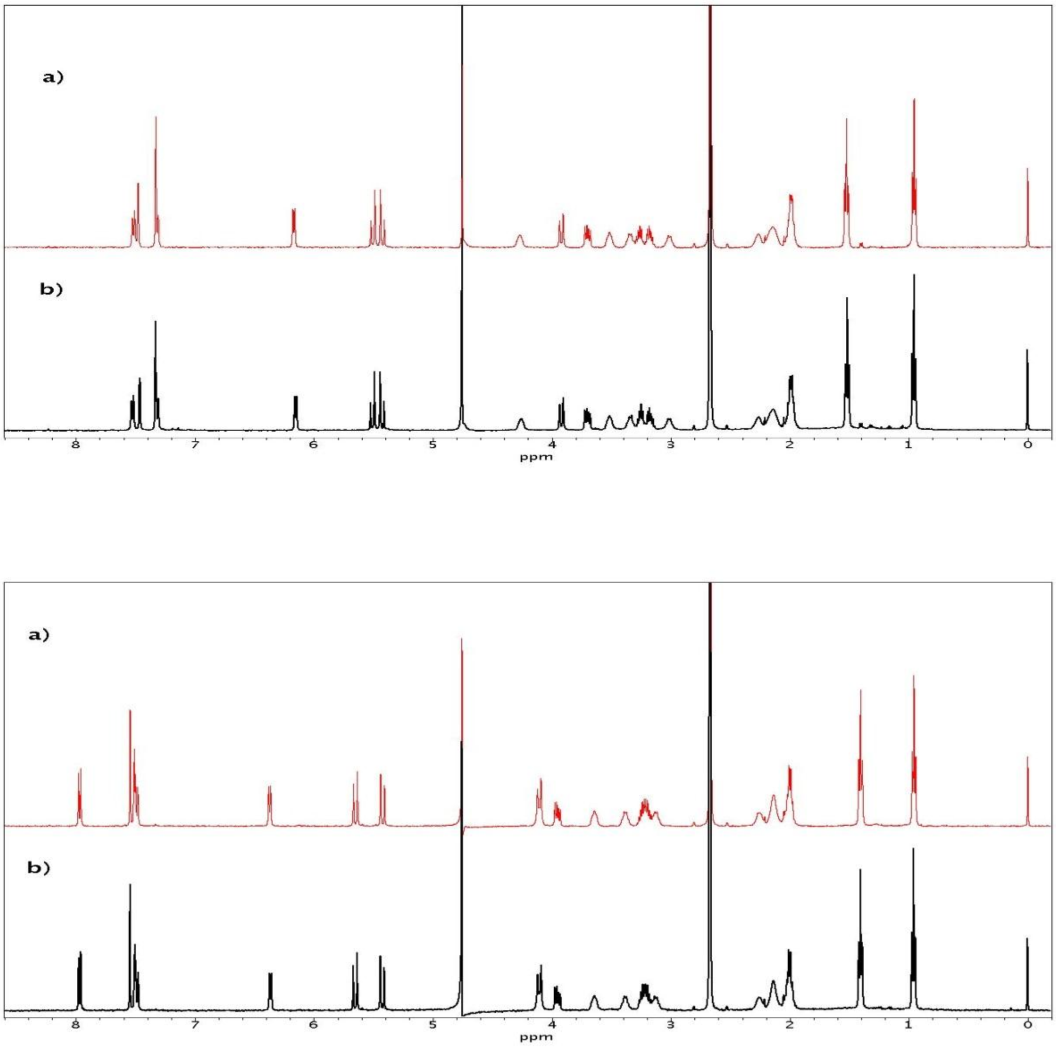


**Figure S2. NMR** analysis of stability test of epimers **1** (upper panel) and **2** (lower panel) in $D_2O$/DMSO-d6, pH 3, temp. 25 °C. In each panel, the red run is for the starting point and the black run is for the end of incubation (after 45 days), showing that the tested compounds 1 and **2** are stable.

**Table S2.** Hydrogen bond (HB) statistic for Compound-1/DNA/Topo I ternary complex (only HB between compound 1 and DNA/TopI receptor are reported).

| Atom names | | MD Family structure 1 | | Docking Family structure 1 | |
|---|---|---|---|---|---|
| Receptor | Compound 1 | HB length [Å] | Population | HB length [Å] | Population |
| ASP 377 OD1, 2 | 20 -O**H** | 2.23 ± 0.54 | 64.35 % | 2.19 ± 0.41 | 24.31 % |
| ARG 208 HH12 | 1-**N** | 2.62 ± 0.30 | 42.84 % | 2.40 +/- 0.31 | 23.86 % |
| LYS 376 HZ1, 2, 3 | 21 >C=**O** | 2.14 ± 0.36 | 23.80 % | 2.13 +/- 0.36 | 19.25 % |
| LYS 376 HZ1, 2, 3 | 21 >**O** | 3.18 ± 0.22 | 7.67 % | 2.88 +/- 0.43 | 10.56 % |
| ARG 208 HE | 20 -**O**H | 2.84 ± 0.28 | 5.53 % | - | - |
| LYS 376 HZ1, 2, 3 | 20 -**O**H | 2.85 ± 0.32 | 4.58 % | - | - |
| THR 562 HG1 | 21 >**O** | 3.32 ± 0.24 | 3.79 % | 3.19 +/- 0.48 | 4.24 % |
| ARG 208 HH21 | 20 -**O**H | 2.31 ± 0.21 | 1.44 % | - | - |
| GLU 200 OE1, 2 | 10 -O**H** | 3.10 ± 0.42 | 1.34 % | 2.55 +/- 0.58 | 4.31 % |
| LYS 269 HZ1, 2, 3 | 10 -**O**H | 2.89 ± 0.45 | 1.17 % | 2.40 +/- 0.57 | 2.94 % |
| ARG 208 HH22 | 1-**N** | 3.42 ± 0.26 | 1.08 % | 3.38 +/- 0.70 | 1.19 % |
| ASN 566 HD21 | 21 >C=**O** | - | - | 2.00 +/- 0.34 | 13.29 % |
| DA 35 O4' | 10 -O**H** | - | - | 2.26 +/- 0.41 | 10.43 % |
| ASN 566 OD1 | 20 -O**H** | - | - | 2.42 +/- 0.50 | 7.38 % |
| DA 35 O5' | 10 -O**H** | - | - | 2.14 +/- 0.45 | 7.19 % |
| DC 34 O3' | 10 -O**H** | - | - | 2.11 +/- 0.35 | 3.52 % |
| TYR 270 H | 10 -**O**H | - | - | 2.07 +/- 0.38 | 2.19 % |
| ARG 208 HH11 | 10 -**O**H | - | - | 2.56 +/- 0.52 | 2.05 % |
| Average H-bond number per residue in Family structure 1 | | | **2.98** | | **2.31** |
| | | MD Family structure 2 | | Docking Family structure 2 | |
| ASN 566 OD1 | 10 -O**H** | 1.84 ± 0.18 | 95.60 % | 2.06 +/- 0.21 | 71.39 % |
| ARG 208 HH21, 22 | 16 >=**O** | 2.11 ± 0.23 | 44.67 % | 2.25 +/- 0.45 | 12.06 % |
| ARG 208 HH12 | 16 >=**O** | 2.28 ± 0.52 | 41.80 % | 2.31 +/- 0.45 | 17.61 % |
| DA 35 N7 | 20 -O**H** | 2.53 ± 0.52 | 29.13 % | 2.86 +/- 0.45 | 3.56 % |
| DA 35 N6 | 20 -O**H** | 2.69 ± 0.40 | 23.60 % | 2.90 +/- 0.94 | 0.56 % |
| DA 35 H62 | 20 -**O**H | 2.70 ± 0.33 | 23.51 % | - | - |
| LYS 269 HZ1, 2, 3 | 21 >C=**O** | 2.17 ± 0.40 | 18.65 % | 2.23 +/- 0.43 | 7.65 % |
| DA 35 H61 | 1-**N** | 3.35 ± 0.19 | 6.87 % | 3.28 +/- 0.63 | 1.67 % |
| LYS 269 HZ1, 2, 3 | 21 >**O** | 3.09 ± 0.27 | 4.65 % | 2.55 +/- 0.57 | 8.31 % |
| ASN 566 HD21 | 10 -**O**H | 2.62 ± 0.41 | 3.68 % | 2.30 +/- 0.45 | 8.89 % |
| DT3 10 O4 | 20 -O**H** | 3.31 ± 0.34 | 1.74 % | 2.22 +/- 0.68 | 0.61 % |
| DG5 11 HO5' | 10 -**O**H | 2.74 +/- 0.53 | 0.15 % | 2.15 +/- 0.48 | 3.44 % |
| THR 562 HG1 | 10 -**O**H | 2.95 +/- 0.97 | 0.04 % | 2.43 +/- 0.58 | 4.06 % |
| TYR 270 H | 21 >C=**O** | - | - | 2.18 +/- 0.38 | 42.83 % |
| DG5 11 O4' | 10 -O**H** | - | - | 2.09 +/- 0.37 | 4.89 % |
| THR 562 OG1 | 10 -O**H** | - | - | 2.24 +/- 0.42 | 4.06 % |
| TYR 270 H | 21 >**O** | - | - | 2.75 +/- 0.54 | 2.78 % |
| Average H-bond number per residue in Family structure 2 | | | **3.85** | | **2.38** |
| | | MD Family structure 3 | | Docking Family structure 3 | |
| DG5 11 N7 | 20 -O**H** | 2.16 ± 0.36 | 86.31 % | 2.74 +/- 0.39 | 8.60 % |
| DG5 11 HO5' | 16 >=**O** | 1.82 ± 0.27 | 66.33 % | 2.03 +/- 0.49 | 19.20 % |

| | | | | | |
|---|---|---|---|---|---|
| DG5 11 O6 | 20 -O**H** | 3.26 ± 0.25 | 40.98 % | 2.68 +/- 0.39 | 12.70 % |
| DG5 11 HO5' | 21 >**O** | 2.24 ± 0.39 | 13.22 % | 2.61 +/- 0.54 | 6.30 % |
| THR 562 HG1 | 16 >=**O** | 2.62 ± 0.81 | 11.89 % | 2.60 +/- 0.74 | 8.15 % |
| THR 591 HG1 | 21 >C=**O** | 2.69 ± 0.66 | 8.64 % | 2.38 +/- 0.74 | 1.15 % |
| GLU 200 OE1, 2 | 10 -O**H** | 2.39 ± 0.53 | 4.40 % | 2.20 +/- 0.61 | 1.50 % |
| DA 35 O4' | 10 -O**H** | 2.16 ± 0.31 | 3.15 % | 2.07 +/- 0.23 | 48.90 % |
| DC 34 H41 | 1-**N** | 3.33 ± 0.23 | 2.50 % | 3.38 +/- 3.38 | 0.05 % |
| THR 591 HG1 | 21 >**O** | 2.96 ± 0.40 | 1.86 % | 2.67 +/- 1.07 | 0.35 % |
| DG5 11 HO5' | 21 >C=**O** | 2.98 ± 0.30 | 1.60 % | 2.35 +/- 0.59 | 2.35 % |
| DA 35 OP2 | 10 -O**H** | 3.24 +/- 0.70 | 0.09 % | 2.50 +/- 0.42 | 4.05 % |
| ARG 208 HH11, 21 | 10 -**O**H | 3.10 +/- 0.76 | 0.05 % | 2.31 +/- 0.35 | 10.15 % |
| ARG 208 HE | 10 -**O**H | 3.19 +/- 1.44 | 0.02 % | 2.76 +/- 0.64 | 5.35 % |
| DA 35 O5' | 10 -O**H** | - | - | 2.65 +/- 0.41 | 20.90 % |
| DC 34 O3' | 10 -O**H** | - | - | 2.13 +/- 0.32 | 20.80 % |
| ASN 196 OD1 | 20 -O**H** | - | - | 2.19 +/- 0.35 | 5.45 % |
| Average H-bond number per residue in Family structure 3 | | | **2.45** | | **1.88** |
| | | MD Family structure 4 | | Docking Family structure 4 | |
| ASN 566 OD1 | 10 -O**H** | 1.81 ± 0.14 | 97.26 % | 1.93 +/- 0.15 | 94.13 % |
| DG5 11 N3 | 20 -O**H** | 2.33 ± 0.43 | 80.24 % | 3.05 +/- 0.28 | 11.81 % |
| ASN 566 HD21 | 10 -**O**H | 2.41 ± 0.25 | 77.41 % | 2.34 +/- 0.31 | 71.56 % |
| ARG 208 HE | 21 >C=**O** | 2.19 ± 0.30 | 25.95 % | 2.24 +/- 0.41 | 16.25 % |
| ARG 208 HH11, 12 | 21 >C=**O** | 2.46 ± 0.45 | 20.07 % | 2.25 +/- 0.37 | 22.09 % |
| ARG 208 HH11 | 20 -**O**H | 2.30 ± 0.28 | 15.72 % | - | - |
| DC 34 O2 | 20 -O**H** | 3.24 ± 0.28 | 11.04 % | 3.00 +/- 1.36 | 0.31 % |
| ARG 208 HH21, 22 | 21 >C=**O** | 2.56 ± 0.45 | 9.75 % | 2.15 +/- 0.53 | 6.00 % |
| DG5 11 O4' | 20 -O**H** | 2.52 ± 0.30 | 5.65 % | 2.38 +/- 0.31 | 10.25 % |
| DG5 11 H22 | 20 -**O**H | 2.68 ± 0.43 | 4.92 % | 2.47 +/- 0.50 | 2.69 % |
| DG5 11 H21 | 21 >**O** | 3.24 ± 0.32 | 4.09 % | 2.66 +/- 0.73 | 1.25 % |
| DG5 11 HO5' | 1-**N** | 2.65 ± 0.44 | 1.68 % | 2.66 +/- 0.55 | 3.56 % |
| DC 34 H41 | 16 >=**O** | 3.18 ± 0.39 | 1.40 % | 3.26 +/- 0.66 | 2.00 % |
| LYS 595 HZ1, 2, 3 | 10 -**O**H | 3.20 ± 0.32 | 0.98 % | 2.94 +/- 0.58 | 3.69 % |
| LEU 565 O | 10 -O**H** | 3.31 +/- 0.31 | 0.53 % | 3.19 +/- 0.46 | 3.44 % |
| ASP 377 OD1, 2 | 20 -O**H** | 2.47 +/- 0.76 | 0.25 % | 2.55 +/- 0.60 | 2.53 % |
| DG5 11 HO5' | 21 >C=**O** | - | - | 2.66 +/- 0.67 | 3.50 % |
| Average H-bond number per residue in Family structure 4 | | | **3.89** | | **2.93** |

| Atom names | | MD Family structure 1 | | Docking Family structure 1 | |
|---|---|---|---|---|---|
| Receptor | Compound 2 | HB length [Å] | Population | HB length [Å] | Population |
| ARG 208 HH12 | 1-**N** | 3.05 ± 0.25 | 32.20 % | 2.56 +/- 0.38 | 19.24 % |
| LYS 376 HZ1, 2, 3 | 21 >C=**O** | 2.21 ± 0.35 | 29.75 % | 2.28 +/- 0.42 | 11.51 % |
| LYS 376 HZ1, 2, 3 | 20 -**O**H | 2.85 ± 0.28 | 9.43 % | - | - |
| GLU 200 OE1, 2 | 10 -O**H** | 2.53 ± 0.64 | 8.05 % | 2.41 +/- 0.52 | 4.71 % |
| DG5 11 HO5' | 16 >=**O** | 2.76 ± 0.69 | 4.56 % | 2.78 +/- 0.58 | 4.38 % |
| ASP 377 OD1, 2 | 20 -O**H** | 3.28 ± 0.36 | 2.29 % | 2.20 +/- 0.41 | 9.22 % |
| LYS 269 HZ1, 2, 3 | 10 -**O**H | 2.82 ± 0.47 | 1.16 % | 2.55 +/- 0.60 | 1.34 % |
| DT3 10 O2 | 20 -O**H** | 2.48 +/- 0.64 | 0.50 % | 2.43 +/- 0.38 | 8.06 % |

| | | | | | |
|---|---|---|---|---|---|
| ASN 566 HD21 | 21 >C=**O** | - | - | 1.96 +/- 0.27 | 28.74 % |
| ASN 566 OD1 | 20 -O**H** | - | - | 2.32 +/- 0.34 | 28.06 % |
| DA 35 O4' | 10 -O**H** | - | - | 2.10 +/- 0.19 | 22.82 % |
| TYR 270 H | 10 -**O**H | - | - | 2.02 +/- 0.22 | 11.50 % |
| ARG 208 HH11 | 10 -**O**H | - | - | 2.64 +/- 0.44 | 7.97 % |
| TYR 270 O | 10 -O**H** | - | - | 1.97 +/- 0.23 | 5.18 % |
| Average H-bond number per residue in Family structure 1 | | | **1.79** | | **2.02** |
| | | MD Family structure 2 | | Docking Family structure 2 | |
| ASN 566 OD1 | 10 -O**H** | 1.98 ± 0.26 | 75.75 % | 2.04 +/- 0.25 | 30.32 % |
| DA 35 N7 | 20 -O**H** | 2.22 ± 0.43 | 45.60 % | 2.73 +/- 0.36 | 12.82 % |
| DA 35 H62 | 20 -**O**H | 2.89 ± 0.35 | 40.15 % | - | - |
| ASN 566 HD21 | 10 -**O**H | 2.68 ± 0.30 | 32.02 % | 2.56 +/- 0.49 | 5.82 % |
| LYS 269 HZ1, 2, 3 | 21 >C=**O** | 2.22 ± 0.42 | 13.53 % | 2.34 +/- 0.51 | 7.14 % |
| DA 35 N6 | 20 -O**H** | 3.07 ± 0.28 | 11.64 % | 2.60 +/- 0.33 | 11.73 % |
| ASN 196 HD22 | 21 >C=**O** | 2.03 ± 0.34 | 10.79 % | 3.34 +/- 0.82 | 0.82 % |
| ARG 208 HH11 | 16 >=**O** | 3.02 ± 0.59 | 7.31 % | 2.16 +/- 0.35 | 13.46 % |
| LYS 595 HZ1, 2, 3 | 10 -**O**H | 2.52 ± 0.40 | 5.92 % | 2.20 +/- 0.59 | 1.71 % |
| LEU 565 O | 10 -O**H** | 3.24 ± 0.20 | 5.72 % | 3.10 +/- 0.69 | 1.09 % |
| DC 34 H42 | 21 >**O** | 2.99 ± 0.34 | 2.62 % | - | - |
| ASN 196 HD22 | 21 >**O** | 3.25 ± 0.25 | 2.16 % | - | - |
| THR 562 OG1 | 10 -O**H** | 2.87 ± 0.50 | 1.81 % | 2.11 +/- 0.32 | 12.00 % |
| LYS 269 HZ1, 2, 3 | 21 >**O** | 2.81 ± 0.43 | 1.46 % | 2.58 +/- 0.58 | 5.02 % |
| DA 35 H61 | 1-**N** | 3.33 ± 0.27 | 1.25 % | 3.29 +/- 0.53 | 2.14 % |
| DG5 11 O4' | 10 -O**H** | 2.38 +/- 0.39 | 0.40 % | 2.25 +/- 0.37 | 8.05 % |
| TYR 270 O | 20 -O**H** | 3.38 +/- 0.63 | 0.12 % | 2.52 +/- 0.44 | 9.23 % |
| THR 562 HG1 | 10 -**O**H | 2.56 +/- 0.66 | 0.11 % | 2.25 +/- 0.42 | 12.32 % |
| TYR 270 H | 21 >C=**O** | - | - | 2.13 +/- 0.42 | 25.82 % |
| DG5 11 HO5' | 10 -**O**H | - | - | 2.21 +/- 0.43 | 14.14 % |
| DA 35 O4' | 20 -O**H** | - | - | 2.07 +/- 0.25 | 11.05 % |
| Average H-bond number per residue in Family structure 2 | | | **3.00** | | **2.12** |
| | | MD Family structure 3 | | Docking Family structure 3 | |
| DA 35 O5' | 10 -O**H** | 2.15 ± 0.27 | 89.60 % | 2.26 +/- 0.42 | 9.53 % |
| DG5 11 O6 | 20 -O**H** | 1.97 ± 0.37 | 87.21 % | 2.41 +/- 0.42 | 4.20 % |
| ASN 566 HD21 | 21 >**O** | 2.49 ± 0.43 | 70.47 % | 3.26 +/- 0.41 | 6.47 % |
| ASN 566 HD21 | 21 >C=**O** | 2.60 ± 0.36 | 42.83 % | - | - |
| DC 34 O3' | 10 -O**H** | 2.37 ± 0.27 | 42.76 % | 2.61 +/- 0.53 | 3.67 % |
| DG5 11 N7 | 20 -O**H** | 2.64 ± 0.56 | 41.81 % | 2.51 +/- 0.37 | 10.67 % |
| DA 35 O4' | 10 -O**H** | 2.94 ± 0.28 | 24.70 % | 2.02 +/- 0.26 | 43.53 % |
| DC 34 H41 | 20 -**O**H | 3.11 ± 0.22 | 12.01 % | - | - |
| LYS 269 HZ1, 2, 3 | 10 -**O**H | 2.28 ± 0.33 | 10.85 % | 2.49 +/- 0.76 | 0.91 % |
| DG5 11 HO5' | 16 >=**O** | 2.13 ± 0.43 | 10.38 % | 2.19 +/- 0.46 | 5.87 % |
| DA 35 OP2 | 10 -O**H** | 2.47 ± 0.42 | 9.17 % | 2.53 +/- 0.64 | 1.73 % |
| LYS 595 HZ1, 2, 3 | 21 >C=**O** | 2.64 ± 0.61 | 3.31 % | 2.36 +/- 0.47 | 4.96 % |
| THR 562 HG1 | 16 >=**O** | 3.43 ± 0.31 | 1.01 % | 2.29 +/- 0.76 | 1.93 % |
| ASN 566 HD21 | 16 >=**O** | 3.31 +/- 0.83 | 0.08 % | 2.98 +/- 0.52 | 8.40 % |
| ARG 208 HH21, 22 | 10 -**O**H | - | - | 2.19 +/- 0.36 | 11.43 % |
| DG5 11 HO5' | 21 >**O** | - | - | 2.68 +/- 0.54 | 11.47 % |
| DG5 11 HO5' | 21 >C=**O** | - | - | 2.37 +/- 0.48 | 8.93 % |

| | | | | | |
|---|---|---|---|---|---|
| ARG 208 HE | 10 -**O**H | - | - | 2.73 +/- 0.55 | 8.20 % |
| TYR 270 O | 10 -O**H** | - | - | 2.33 +/- 0.48 | 7.13 % |
| TYR 270 H | 10 -**O**H | - | - | 2.30 +/- 0.41 | 7.07 % |
| ARG 208 HH11, 12 | 10 -**O**H | - | - | 2.78 +/- 0.48 | 6.27 % |
| Average H-bond number per residue in Family structure 3 | | | **4.75** | | **1.92** |
| | | MD Family structure 4 | | Docking Family structure 4 | |
| ASN 566 OD1 | 10 -O**H** | 1.82 ± 0.15 | 97.39 % | 1.92 +/- 0.15 | 80.48 % |
| DG5 11 N3 | 20 -O**H** | 2.05 ± 0.30 | 78.28 % | 2.82 +/- 0.27 | 23.09 % |
| ASN 566 HD21 | 10 -**O**H | 2.43 ± 0.24 | 53.29 % | 2.24 +/- 0.29 | 57.91 % |
| ARG 208 HH11 | 21 >C=**O** | 2.20 ± 0.34 | 44.80 % | 2.17 +/- 0.39 | 39.22 % |
| ARG 208 HE | 21 >C=**O** | 2.42 ± 0.46 | 17.25 % | 2.26 +/- 0.39 | 19.91 % |
| DG5 11 HO5' | 20 -**O**H | 2.09 ± 0.27 | 16.82 % | - | - |
| DC 34 O2 | 20 -O**H** | 2.77 ± 0.54 | 15.95 % | 2.72 +/- 0.50 | 7.96 % |
| ARG 208 HH21 | 21 >C=**O** | 2.53 ± 0.47 | 14.32 % | 2.28 +/- 0.44 | 14.57 % |
| DG5 11 O4' | 20 -O**H** | 2.88 ± 0.30 | 12.24 % | 2.34 +/- 0.40 | 3.83 % |
| ARG 208 HH11 | 20 -**O**H | 2.17 ± 0.28 | 6.48 % | - | - |
| ARG 208 HE | 20 -**O**H | 2.53 ± 0.39 | 4.95 % | - | - |
| DG5 11 HO5' | 1-**N** | 2.44 ± 0.34 | 4.38 % | 2.33 +/- 0.44 | 2.96 % |
| DG5 11 H22 | 20 -**O**H | 2.68 ± 0.38 | 3.63 % | - | - |
| DC 34 H42 | 16 >=**O** | 2.99 ± 0.37 | 3.52 % | 3.22 +/- 0.80 | 1.00 % |
| ARG 208 HH21 | 20 -**O**H | 2.73 ± 0.44 | 2.92 % | - | - |
| ARG 206 HH12, 22 | 21 >C=**O** | 2.20 ± 0.43 | 2.86 % | 2.35 +/- 0.50 | 1.43 % |
| DG5 11 H21 | 21 >**O** | 3.21 ± 0.33 | 1.58 % | 2.07 +/- 0.75 | 0.35 % |
| ARG 206 HH22 | 21 >**O** | 3.02 ± 0.28 | 1.42 % | 3.04 +/- 0.65 | 1.26 % |
| LYS 595 HZ1, 2, 3 | 10 -**O**H | 3.01 +/- 0.45 | 0.87 % | 3.00 +/- 0.62 | 1.65 % |
| DG5 11 H21, 22 | 21 >C=**O** | 2.46 +/- 0.29 | 0.78 % | 3.00 +/- 0.41 | 4.89 % |
| TYR 270 H | 21 >**O** | - | - | 2.52 +/- 0.42 | 3.13 % |
| Average H-bond number per residue in Family structure 4 | | | **3.89** | | **2.73** |
